# Clear Cell Papulosis: A Rare Pediatric Dermatosis

**DOI:** 10.1155/2018/3908505

**Published:** 2018-08-12

**Authors:** Keng Wein Jeanette Tan, Jin Ho Chong, Jean Aan Mark Koh

**Affiliations:** ^1^Department of Pediatric Medicine, KK Women's and Children's Hospital, Singapore; ^2^Department of Dermatology, KK Women's and Children's Hospital, Singapore

## Abstract

The diagnosis and management of pediatric hypopigmented lesions can be challenging given their wide range of differentials. In this case report, we present a case of a 3-year-old Chinese boy who was initially treated for tinea versicolor but subsequently diagnosed to have clear cell papulosis. The features, diagnosis, and management of clear cell papulosis are discussed in this article to raise awareness of this condition amongst pediatricians.

## 1. Introduction

Clear cell papulosis (CCP) is a benign childhood skin condition. Its presentation can be easily misdiagnosed. It is thus important for pediatricians to be aware of CCP and to consider this diagnosis, especially when initial treatment yields no improvement.

## 2. Case Presentation

**Figure 1:**
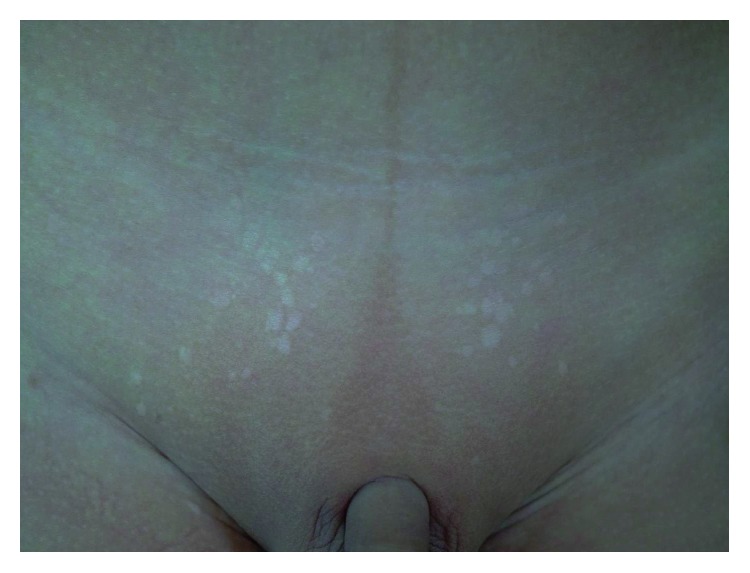
Multiple subcentimeter hypopigmented macules located in the lower abdomen and suprapubic region.

A healthy 3-year-old Chinese boy presented with multiple asymptomatic hypopigmented subcentimeter macules over his lower abdomen and suprapubic region (Figure 1). There was no preceding trauma or inflammation. The rest of his skin examination was normal. There was no family history of similar lesions.

He was initially diagnosed with tinea versicolor. However, after a course of topical antifungal cream, the lesions remained unchanged in number, size, and appearance. Upon review by a dermatologist, he was diagnosed with clear cell papulosis. No further investigations or treatment were needed. His parents were reassured, and he was discharged from follow-up.

## 3. Discussion

Clear cell papulosis (CCP) is an uncommon condition with characteristic clinical and histopathological findings. It was first described in 2 young brothers from Taiwan by Kuo et al. [1]. Since then, there have been about 36 subsequent reports [2].

CCP occurs in early childhood, in children less than 6 years. There has only been one case reported in an adult [3]. It has a female predominance. Reports of the siblings with the condition suggest a possible genetic predisposition with an autosomal recessive inheritance given that none of the parents recall having similar skin lesions during their childhood [1, 4, 5].

Clinically, CCP is characterized by multiple asymptomatic hypopigmented macules or flat papules found mainly on the lower aspect of the abdomen or along the mammary lines. The back, buttocks, and extremities are seldom involved. The distribution of the lesions is almost always bilateral but not necessarily symmetrical. Each macule is small, less than 1 cm. The number of lesions increases over the first few months to years, ranging from 2 to over 100.

CCP can be distinguished from other pediatric hypopigmented dermatoses including postinflammatory hypopigmentation, vitiligo, nevus depigmentosus, tinea versicolor, and hypopigmented mycosis fungoides based on its features: its restricted distribution, size, and number of the lesions, as well as its asymptomatic nature. In this particular case, the presentation, progress, and clinical features are classical of CCP. The age of presentation and lack of response to antifungal treatment make the diagnosis of tinea versicolor very unlikely. Moreover, the lesions are non-scaly and asymptomatic.

In cases where there is uncertainty, a skin biopsy can be done to confirm the diagnosis. The hallmark histological finding of CCP is the proliferation of large clear cells within the basal epidermis. These cells exist singly or more rarely in small clusters. They are larger than adjacent keratinocytes and do not show features of malignancy such as cellular atypia, nuclear pleomorphism, hyperchromasia, and abnormal mitoses. Other features include mild acanthosis, mild hyperkeratosis, and decreased basal melanin. Histochemical and immunohistochemical studies from all reports of CCP show positivity in mucin, carcinoembryogenic antigen (CEA), epithelial membrane antigen (EMA), cytokeratin (CK) AE1 and/or AE3, gross cystic disease fluid protein-15, cell adhesion molecule-5.2, CK-7, and colloidal iron and are negative for S100 [6, 7].

Toker cells, precursors of Paget's disease, have been postulated to be the cell of origin in CCP due to their similar anatomical distribution along the mammary line, histological features, and immunohistochemial profiles with both staining positively for EMA, CK-7, and other low-molecular weight cytokeratins. Toker cells, however, stain negatively for CEA and mucin. More research is needed to investigate the exact cell of origin [5, 8].

CCP requires no investigation or treatment. The lesions will self-resolve in early to late childhood. In the largest case series to date, Tseng et al. demonstrated that spontaneous regression of the lesions was seen in approximately 86% of patients after a median follow-up of 11.5 years; 64.3% showed a reduction in the lesion count, while 21.4% showed complete resolution. None of the patients progressed to Paget's disease [6].

## 4. Conclusion

CCP is an uncommon but distinctive benign childhood dermatosis. It requires no intervention with spontaneous resolution occurring in childhood. A skin biopsy can be performed if uncertain or in atypical cases to confirm the diagnosis.

## Abbreviations

CCP: Clear cell papulosis.
